# Arteriovenous cerebral blood flow correlation in moderate-to-severe traumatic brain injury: CT perfusion study

**DOI:** 10.1016/j.bas.2023.102675

**Published:** 2023-09-21

**Authors:** Alex O. Trofimov, Darya I. Agarkova, Kseniia A. Trofimova, Edwin M. Nemoto, Olga A. Bragina, Denis E. Bragin

**Affiliations:** a Department of Neurological Diseases, Privolzhsky Research Medical University, Nizhny Novgorod, Russia; b Department of Neurology, University of New Mexico School of Medicine, Albuquerque, USA; c Lovelace Biomedical Research Institute, Albuquerque, NM, USA

**Keywords:** CBF, Traumatic brain injury, PCT

## Abstract

**Introduction:**

The relationship between arterial and venous blood flow in moderate-to-severe traumatic brain injury (TBI) is poorly understood.

**The research question:**

was to compare differences in perfusion computed tomography (PCT)-derived arterial and venous cerebral blood flow (CBF) in moderate-to-severe TBI as an indication of changes in cerebral venous outflow patterns referenced to arterial inflow.

**Material and methods:**

Moderate-to-severe TBI patients (women 53; men 74) underwent PCT and were stratified into 3 groups: I (moderate TBI), II (diffuse severe TBI without surgery), and III (severe TBI after the surgery). Arterial and venous CBF were measured by PCT in both the internal carotid arteries (CBFica) and the confluence of upper sagittal, transverse, and straight sinuses (CBFcs).

**Results:**

In group I, CBFica on the left and right sides were significantly correlated with each other (p < 0.0001) and with CBFcs (p = 0.048). In group II, CBFica on the left and right sides were also correlated (P < 0.0000001) but not with CBFcs. Intracranial pressure reactivity (PRx) and CBFcs were correlated (p = 0.00014). In group III, CBFica on the side of the removed hematoma was not significantly different from the opposite CBFica (P = 0.680) and was not correlated with CBFcs.

**Discussion and conclusion:**

The increasing severity of TBI is accompanied by a rising uncoupling between the arterial and venous CBF in the supratentorial vessels suggesting a shifting of cerebral venous outflow.

## Highlights

• The increasing traumatic brain injury severity is accompanied by a rising uncoupling between the arterial and venous cerebral blood flow.

## 1 Introduction

The concept of cerebral microcirculation evaluation based on the axis-voxel analysis of brain tissue X-ray density changes during the passage of contrast agent through the cerebrovascular bed by perfusion computed tomography (PCT) was proposed by Axel L. in 1980 (Axel, 1980). The use of PCT in the routine treatment of cerebral strokes has achieved revolutionary results. However, the potential of PCT is still underutilized, although it has been employed in acute stroke prediction (Zahn et al., 2023; Zhang et al., 2023). One such possibility is to assess the volumetric characteristics not only of the microcirculatory bed but also the blood flow in large vessels: arteries, veins, and brain sinuses (Tao et al., 2022). This is crucial for cerebral blood flow (CBF) studies because other techniques (ultrasonic Doppler, duplex, triplex systems, etc.) only operate with speed and resistive characteristics (Chandrapatham et al., 2021). Ultrasound Doppler studies have shown correlations between the velocities in the main arteries and veins in healthy individuals, but these correlations were impaired in severe TBI (.). Thus, to date, the relationship between arterial and venous blood flow in moderate-to-severe TBI is poorly understood. The aim of this study was to assess the differences in PCT-derived arterial and venous CBF in patients with moderate-to-severe TBI and after surgical removal of intracranial hematomas. We hypothesized that an increase in the severity of TBI correlates with arterial and venous CBF uncoupling.2 Methods.

### 1.1 Study design and population

This non-randomized retrospective single-center study was conducted as an analysis of a prospectively maintained database cohort (2013–2022). The study protocol was approved by the Regional Clinical Hospital named Local Ethical Committee. Inclusion criteria were as follows: moderate and severe TBI within 6 h after injury, Glasgow Coma Scale (GCS) less than 13, multiphase PCT follow-up for at least 12 h, and available mortality data. Exclusion criteria were as follows: less than 16 and more than 75 years old (yo), serum blood creatinine level more than 120 mg/l, and GCS 3 and more than 12.127 moderate-to-severe TBI patients (w 53; m 74) were stratified into 3 groups: group I (moderate TBI, 18–59 years old (yo), n = 49); group II (severe diffuse TBI without the intracranial hematoma, 19 to 56 yo, n = 47); and group III (severe TBI after the intracranial surgery, 19 to 58 yo, n = 31).

### 1.2 Perfusion computed tomography

All patients underwent PCT 1–2 days after TBI using a 64-slice Philips Ingenuity CT (Philips Medical Systems, Cleveland, USA). The PCT examination included an initial non-contrast CT of the brain. Extended scanning was further performed in 16 “areas of interest”, covering 160 mm in the z-axis within 60 s, with the administration of a contrast agent (“Perfusion JOG” mode). The scanning parameters were 80 kVp, 150 mA, effective dose = 3.3 mSv, slice thickness = 5 mm, and collimation = 64 × 0.625 mm. A total of 50 mL of contrast agent Ultravist 370 (Schering, Germany) was administered with a syringe injector Stellant (Medrad, PA, USA) into the right cubital vein through a standard catheter (20G) at a rate of 5 ml/s. The acquired data were transferred to the Philips Extended Brilliance Workspace (Philips Health Care, Best, the Netherlands). Artery and vein marks were automatically recorded, followed by a cluster-analysis algorithm controlled manually by adjusting indices in the time-concentration plot. Perfusion maps were derived using the Bayesian probabilistic method from the tissue time-attenuation curve, which is linearly related to iodine concentration on an aper-voxel basis over time. Quantitative PCT parameters of CBF, as well as other parameters (cerebral blood volume, mean transit time, time-to-peak), were calculated on a voxel-wise basis and were used to generate color-coded maps. Regions of interest were established in the projection of both terminal segments (C7) of the internal carotid arteries (ICA) and in the projection of the confluence of the upper sagittal, transverse, and straight sinuses (*confluens sinuum - CS)*. Thus, we measured СBF directly inside both the internal carotid arteries and the CS.

### 1.3 Intracranial pressure and pressure-reactivity index monitoring

ICP was monitored using the parenchymal probe (Codman MicroSensors ICP, Codman & Shurtleff, Raynham, USA). In diffuse TBI cases, ICP probes were inserted into the parenchyma of the non-dominant frontal lobe. In the surgical case, the ICP probes were placed in lesion-side white matter. Physiological parameters and ICP were continuously recorded every 3 s during the PCT using a bedside monitor (IntelliView MP5, Philips Medizin Systeme, Germany). The pressure reactivity index (PRx) was calculated from the measured parameters as described earlier (Cardim et al., 2020).

### 1.4 Statistical analysis

The data are presented as mean [interquartile range] for continuous variables. Fisher's exact test was used for categorical variables. Statistical analysis of continuous variables was done by Wilcoxon signed-rank test (T-criterion Wilcoxon) as appropriate. The nonparametric Spearman rank correlation coefficients (R) were used to assess correlations between the variables. The significance level was set at p < 0.05. Statistical analysis was done using Statistica 12 (TIBCO Software Inc., Palo Alto, USA).

## 2 Results

**Fig. 1:**
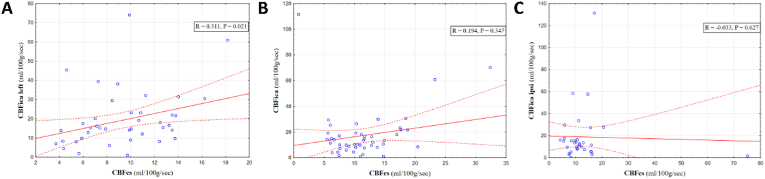
Cerebral blood flow in confluens sinuum (CBFcs) plotted against cerebral blood flow in the left internal carotid artery (CBF_ICA left_) in Group 1 (**A**), in Group 2 (**B**), and in the ipsilateral side of removal intracranial hematoma in Group 3 (CBF_ICA ipsi_) (**C**). Dashed red lines represent 95% confidence intervals for the regression (solid red line).

The obtained data are summarized in Table 1, Table 2 and in Fig. 1. In group I (moderate TBI), there were no significant differences between CBF in the internal carotid arteries on the left and right side (Z = 2.330; P = 0.237). The left and right CBFica were significantly correlated with CBFcs (R = 0.311, P < 0.05, R = 0.298, P < 0.05, respectively). ICP in this group was not monitored. In group II (severe diffuse TBI without the intracranial hematoma), the left and right CBFica were also similar (Z = 2.027; P = 0.651). However, there was no correlation between CBFica and CBFcs (P > 0.05). A significant correlation was observed between PRx and CBFcs (R = 0.628, P = 0.00014). A correlation between PRx and both CBFica was almost significant (p = 0.112 and p = 0.137). In group III (after the TBI surgery) CBFica on the side of the removed hematoma was not significantly different from CBFica on the opposite side (Z = 1.289; P = 0.059). Moreover, there was no correlation between both CBFica and CBFcs (on the side of removed hematoma, R = - 0.033. P = 0.627; on the opposite side, R = - 0.021, P = 0.521).

**Table 1:** Comparison of the obtained CBF-values in the internal carotid arteries and in the confluence of upper sagittal, transverse and straight sinuses.

	I CBFica left (ml/100 g/s)	II CBFica right (ml/100 g/s)	III CBFcs (ml/100 g/s)	P (I-II)	P (I-III)	P (II-III)
Group 1 N = 49	17.27 [11.62–24.32]	16.33 [10.29–22.74]	10.52 [8.51–12.89]	0,237	0.016*	0.021*
Group 2 N = 47	15.23 [9.46–22.95]	16.85 [12.13–21.33]	10.71 [7.51–13.69]	0.651	0.347	0.432
Group 3 (ipsilateral and contralateral side former hematoma, respectively) N = 31	17.21 [14.46–20.12]	19.84 [16.33–24.21]	10.59 [8.31–15.12]	0.059	0.627	0.521

CBFica –cerebral blood flow in internal carotid artery, CBFcs - cerebral blood flow in confluence of upper sagittal, transverse and straight sinuses, data are presented as mean values [inter-quartile range], * – р < 0.05.

**Table 2:** Comparison of the obtained values of GCS, ICP and PRx values.

		GCS	ICP (mmHg)	PRx
1	Group 1 N = 49	12.5 (Dobrzeniecki et al., 2018; Takahashi et al., 1994)	–	–
2	Group 2 N = 47	10 (Westermaier et al., 2014; van der Zijden et al., 2022; Mills et al., 2013)	22 [10.5–33.4]	0,33 [0.23–0.44]
3	Group 3 (ipsilateral and contralateral side former hematoma) N = 31	9 (Cardim et al., 2020; Westermaier et al., 2014; van der Zijden et al., 2022; Mills et al., 2013)	25 [9.5–33.5]	0,38 [0,10–0,56]
	Р (1–2)	0,00001 *	–	–
	Р (1–3)	0,00001 *	–	–
	Р (2–3)	0.06	0.078	0.048*

GCS -Glasgow Coma Score, ICP – intra-cranial pressure, PRx – pressure reactivity index, data are presented as mean values [inter-quartile range], * – р < 0.05.

We found a significant correlation between PRx and CBFcs (R = 0.485, P = 0.002). However, there was no significant correlation between PRx and both CBFica (p > 0.05). In group III, we found a statistically significant correlation between the level of wakefulness by GCS and CBFcs (R = 0.349, P = 0.046), which was not observed in groups I and II. A two-tailed Fisher test demonstrated that arterial-venous CBF uncoupling was frequently observed in PRx >0.3 and ICP >21 mmHg patients (P < 0.0001).

## 3 Discussion

Multiphase PCT allows the estimation of the blood volume flowing through a given region of interest per unit of time (i.e., CBF in ml/100 g/s), rather than just measuring blood flow velocity (cm/sec) as in transcranial Doppler (Cardim et al., 2020). In contrast to “blind” TCD and even duplex or triplex devices, dynamic multiphase PCT enables the direct visualization of all major cerebral vessel segments and the measurement of CBF in specific intracranial segments with high accuracy (Cardim et al., 2020). PCT-derived CBF data do not depend on the angle of insonation errors, leading to more accurate measurements of CBF, approaching the accuracy of multiplanar rotational angiographic settings (.). Cardim et al. showed significant correlations between TCD-derived CBF velocity of USS and both hemispheric blood flow in healthy volunteers; however, these correlations do not consistently persist in TBI patients (.). Furthermore, the arteriovenous correlation between volume velocity in large arterial (both ICAs) and venous (USS) vessels in moderate-to-severe TBI has not been investigated. We measured instantaneous values of volume flow in both ICA and USS using PCT instead of time-averaged TCD-derived values obtained with conventional ‘blind’ TCD devices, and, to our knowledge, this study is the first to use dynamic multiphasic PCT to quantify CBF in large cerebral vessels in patients and document shifts in venous outflow patterns after severe TBI correlated with the outcome (Westermaier et al., 2014).

Using this technique, we correlated CBF in USS and both ICA in various groups of moderate-to-severe TBI patients and found that the increasing TBI severity is accompanied by a growing uncoupling between the arterial and venous CBF in the supratentorial vessels. One possible reason for the discordance in the arteriovenous correlation of CBF may be changes in cerebral vascular compliance in TBI. An increase in arterial compliance in TBI has been previously shown, especially after the removal of intracranial hematomas. The cause may be edema of the arterial wall and endothelial dysfunction, leading to an increase in the stiffness of the arterial wall (van der Zijden et al., 2022). The walls of the veins and cerebral sinuses can be compressed by brain edema (Mills et al., 2013), making cerebral veins and sinuses more susceptible than arteries to tissue edema and ICP rise. Changes in large vessel wall stiffness with increasing TBI severity may not be proportional, leading to alterations in arterial and venous CBF correlation, consistent with previous research (Dobrzeniecki et al., 2018).

Another possible reason for the alteration in arterial-venous correlation might be increased cerebrovascular resistance. While a large body of data exists on the growth in arterial and arteriolar resistance in various types of TBI (Takahashi et al., 1994; Chen et al., 2015), changes in venous resistance after head injury remain poorly understood. It is known, however, that a modification in the vessel lumen from circular to any other configuration can increase vascular resistance (Shimamura and Ohkuma, 2014; Eckert, 2021). It has been suggested that capillary compression in the region where the hematoma was removed could obstruct microcirculation blood flow (Dewey, 1974; Sharples et al., 1995), potentially leading to a decrease in the number of functioning capillaries (i.e., capillary rarefaction) and increased cerebrovascular resistance. Under these conditions, temporary microvascular shunts might open to maintain perfusion in the locus of the hematoma (Daley and Narayanan, 2010). All these processes further exacerbate the unevenness of the CBF and, therefore, alter the correlations between arterial and venous blood flow in TBI.

Although our study examines a relatively large cohort, several limitations remain. First, bone artifacts significantly limit the assessment of subtentorial CBF by PC, and we were unable to study CBF in the main arteries and veins in those brain regions. Second, the study design was cross-sectional, so the lack of a CBF-age correlation in moderate-to-severe TBI requires further validation. While the venous region of interest was near to *confluens sinuum,* we cannot completely eliminate a mathematical error associated with ROI space measurements. 5 Conclusion.

The increasing severity of TBI is accompanied by the uncoupling of arterial and venous CBF in the supratentorial vessels. These findings suggest a shift in venous outflow patterns with increased ICP after severe TBI, which may be related to shifts in venous vascular compartments due to brain edema, reduced cerebral vascular compliance, and increased cerebrovascular resistance. Further studies are needed to identify the mechanisms behind the development of this mismatch.

## Declaration of competing interest

The authors declare that they have no conﬂict of interest.
